# Metagenomic Analyses of Microbial and Carbohydrate-Active Enzymes in the Rumen of Holstein Cows Fed Different Forage-to-Concentrate Ratios

**DOI:** 10.3389/fmicb.2019.00649

**Published:** 2019-03-29

**Authors:** Lijun Wang, Guangning Zhang, Hongjian Xu, Hangshu Xin, Yonggen Zhang

**Affiliations:** College of Animal Science and Technology, Northeast Agricultural University, Harbin, China

**Keywords:** holstein cow rumen, metagenomics, microbiome, CAZymes, taxonomic diversity

## Abstract

The objectives of this study were to investigate the effects of different forage-to-concentrate ratios and sampling times on the genetic diversity of carbohydrate-active enzymes (CAZymes) and the taxonomic profile of rumen microbial communities in dairy cows. Six ruminally cannulated Holstein cows were arbitrarily divided into groups fed high-forage (HF) or low-forage (LF) diets. The results showed that, for glycoside hydrolase (GH) families, there were greater differences based on dietary forage-to-concentrate ratio than sampling time. The HF treatment group at 4 h after feeding (AF4h) had the most microbial diversity. Genes that encode GHs had the highest number of CAZymes, and accounted for 57.33% and 56.48% of all CAZymes in the HF and LF treatments, respectively. The majority of GH family genes encode oligosaccharide-degrading enzymes, and GH2, GH3, and GH43 were synthesized by a variety of different genera. Notably, we found that GH3 was higher in HF than LF diet samples, and mainly produced by *Prevotella*, *Bacteroides*, and unclassified reads. Most predicted cellulase enzymes were encoded by GH5 (the BF0h group under HF treatment was highest) and GH95 (the BF0h group under LF treatment was highest), and were primarily derived from *Bacteroides*, *Butyrivibrio*, and *Fibrobacter.* Approximately 67.5% (GH28) and 65.5% (GH53) of the putative hemicellulases in LF and HF treatments, respectively. GH28 under LF treatment was more abundant than under HF treatment, and was mainly produced by *Ruminococcus*, *Prevotella*, and *Bacteroides*. This study revealed that HF-fed cows had increased microbial diversity of CAZyme producers, which encode enzymes that efficiently degrade plant cell wall polysaccharides in the cow rumen.

## Introduction

Cellulose and hemicellulose in plant cell wall polysaccharides are the most abundant renewable resources in nature, and the development and use of these compounds is considered one of the most effective ways to alleviate energy problems, such as fossil fuels being a finite resource that produce pollution (Walia et al., 2017). The rumen is recognized as a natural bioreactor for highly efficient structural carbohydrates (e.g., cellulose and hemicellulose) degradation (Codron and Clauss, 2010) because it has a large number of microorganisms that can degrade cellulose. Moreover, the cellulase produced by microorganisms is usually considered safe, stable, and efficient for cellulose degradation (Bickhart and Weimer, 2017). Cellulose-degrading microorganisms in the rumen mainly include bacteria, fungi, and protozoa (Pope et al., 2012). Bacteria account for approximately 95% of all microorganisms (Mackie et al., 2000), and, unsurprisingly, they play a critical role in cellulose decomposition during rumen fermentation (Pang et al., 2017). Owing to the presence of numerous fiber-degrading microorganisms and enzymes, 60–65% of structural carbohydrates can be degraded within 48 h of fermentation by various microorganisms that provide nutrients for host ruminant growth and development (Wang and Duan, 2014).

Rumen microorganisms produce a series of enzymes known as carbohydrate-active enzymes (CAZymes) that can break down plant cell walls. There are four types of CAZymes that are distinguished based on protein sequence, gene sequence, and structural similarities: glycoside hydrolases (GHs), glycosyltransferases (GTs), polysaccharide lyases (PLs), and carbohydrate esterases (CEs); these CAZymes cooperatively contribute to dietary cellulose, hemicellulose, and pectin deconstruction (Lim et al., 2013; Kala et al., 2017). Furthermore, related non-enzymatic species are known as carbohydrate-binding modules (CBMs). Studies have shown that CBMs can increase the catalytic efficiency of enzymes by specifically binding polysaccharides and increasing enzyme concentration (Boraston et al., 2004; Jones et al., 2018).

Cellulose is the most important component in ruminant diets and is essential for rumen fermentation, and the rumen has developed into an effective and complex cellulose degradation system (Fox et al., 1992; Buchanan et al., 2010). This process has been the focus of metagenomic research aimed at identifying and capturing the diversity of enzyme activity. Many metagenomic studies have reported CAZyme diversity in different ruminants, such as Holstein–Friesian crossbred steers (Jose et al., 2017a), Angus cattle (Brulc et al., 2009), buffalo (Patel et al., 2014), and Saudi sheep (Almasaudi et al., 2017). Nine endoglucanases, 12 esterases, and one cyclodextrinase were detected from the rumen metagenomic library of dairy cows (Ferrer et al., 2010). Subsequently, several specific polysaccharide-degrading enzymes were isolated from the rumen using metagenomics techniques (Math et al., 2010; Huoqing et al., 2011). Additionally, most research has been conducted on CAZymes and digestive microbiota, but few studies have evaluated the effects of different forage-to-concentrate ratios and sampling time on the genetic diversity of CAZymes and taxonomic profile of rumen microbial communities in dairy cows. Therefore, this study was designed and carried out to explore CAZyme diversity and characteristics under different forage-to-concentrate ratios and sampling times, and identify the microbes that produce CAZymes.

## Materials and Methods

This study was carried out in accordance with the regulations of Instructive Notions with Respect to Caring for Experimental Animals, Ministry of Science and Technology of China. The protocol was approved by the Ethical Committee of the College of Animal Science and Technology of Northeast Agricultural University.

### Experimental Design, Animals Feeding, and Sample Collection

Six ruminally cannulated Holstein cows that averaged 3.2 ± 0.70 (mean ± SE) years of age were used in this experiment. Cows were housed in individual tie stalls. The treatments contained 70% (high-forage, HF) and 30% (low-forage, LF) dietary forage (dry matter basis), respectively. For 3 weeks before sampling, animals were fed once daily at 8:00 AM and allowed *ad libitum* consumption of 110% of their expected intake. The ingredient and nutritional composition of the two diets are presented in Table 1. Rumen content samples were collected before feeding (i.e., at 0 h, BF0h) and 4 h after feeding (AF4h) via a ruminal fistula. Collective representative samples of ruminal contents from each animal were extruded through four layers of cheesecloth. One part of each homogenized pellet was mixed with RNAlater (Ambion, Austin, TX, United States), which is a reagent that protects and stabilizes bacterial RNA, and the rest of each pellet was used for DNA extraction and enzyme activity determination. All samples were placed in liquid nitrogen within 5 min, and then taken to the laboratory and stored at -80°C until further testing.

**Table 1:** Ingredients and nutritional composition of experimental diets.

Item	Dietary concentrate- to-forage ration	
	30:70	70:30
Ensiled maize stalks	57.0	19.0
Chinese ryegrass hay	4.0	6.0
Alfalfa pellets	9.0	5.0
Steam-flaked maize	13.0	54.0
Soybean curb residue	15.2	13.7
Mineral mix1	1.8	2.3
**Nutrition composition**		
ME2 (MJ/kg)	9.07	11.70
CP (%DM)	12.7	12.7
NDF (%DM)	54.2	35.3
ADF (%DM)	25.3	19.2
Starch (%DM)	12.2	40.9
Ca (%DM)	0.64	0.63
P (%DM)	0.32	0.33

*(1) ME, metabolizable energy; CP, crude protein; NDF, neutral detergent fiber; ADF, acid detergent fiber. (2) Mineral mix contained 18.50% Ca, 6.00% P, 4.2% Mg, 1.4% K, 2.6% S, 7.5% Na, 12.0% Cl, 30 mg/kg of Se, 0.25% Zn, 0.25% Fe, 0.25% Mn, 1,100 mg/kg of Cu, 15 mg/kg of I, 265,000 IU/kg of vitamin A, 110,200 IU/kg of vitamin D, and 2,300 IU/kg of vitamin K*.

### Enzyme Activity Analysis

For the enzyme activity assay, frozen pellets were thawed at room temperature. After being centrifuged at 3000 *g* for 10 min (4°C), 10–15 mL of supernatant was taken for sonication (power, 400 W; crushed three times for 30 s each time at 30 s intervals), and the crushed liquid was subsequently tested. The assayed CMCase, β-glucosidase, xylanase, and β-xylosidase activity was measured using the 3,5-dinitrosalicylic acid method (Miller et al., 1960; Yang and Xie, 2010).

### RNA Extraction, RNA Reverse Transcription, and qPCR Primer Design and Analysis

RNA extraction was performed using the liquid nitrogen grinding method and TRIzol reagent (Ambion, Carlsbad, CA, United States) following the protocols described by Kang et al. (2009) with some modifications. The RNA was reverse-transcribed into cDNA using a PrimeScript^TM^ 1st strand cDNA Synthesis Kit (Code No. 6110A, Takara, Dalian, China), following the kit instructions. The reverse-transcribed PCRs were conducted as follows: 37°C for 15 min, 85°C for 5 s, and 4°C for 10 min. The cDNA was stored at -80°C. The PCR primers used are listed in Table 2 and were assembled based on previous literature (Khafipour et al., 2009; Edwards et al., 2010). Primers were provided by Sangon Biotech Co., Ltd., (Shanghai, China). The Real-Time qPCR performed using Takara SYBR^®^ Premix Ex Taq^TM^ Synthesis Kit (Code No. RR420A, Takara, Dalian, China), following the kit instructions. Abundance of these microbes were expressed as a proportion of total estimated rumen bacterial 16S rDNA according to the equation: relative quantification = 2^-(Cttarget-Cttotalbacteria)^, where Ct represents threshold cycle (Guo et al., 2010).

**Table 2:** Primers used for real-time PCR quantification.

Target bacteria	Primer	Tm size	Product	References
		(°C)	(bp)	
General Bacteria 16Sr DNA	F: 5′- CGGCAACGAGCGCAACCC-3′	58	130	Egan, 2005
	R: 5′-CCATTGTAGCACGTGTGTAGCC-3′			
*Ruminococcus albus*	F: 5′-CCCTAAAAGCAGTCTTAGTTCG-3′	54	176	Khafipour et al., 2009
	R: 5′-CCTCCTTGCGGTTAGAACA-3′			
*Ruminococcus flavefaciens*	F: 5′-CGAACGGAGATAATTTGAGTTTACTTAGG-3′	58	132	Khafipour et al., 2009
	R: 5′-CGGTCTCTGTATGTTATGAGGTATTACC-3′			
*Fibrobacter succinogenes*	F: 5′-GGAGCGTAGGCGGAGATTCA-3′	59	97	Khafipour et al., 2009
	R: 5′-GCCTGCCCCTGAACTATCCA-3′			
*Butyrivibrio fibrisolvens*	F: 5′-ACCGCATAAGCGCACGGA-3′	59	124	Khafipour et al., 2009
	R: 5′-CGGGTCCATCTTGTACCGATAAAT-3′			

### DNA Extraction, 16S rRNA Gene Amplicon Preparation, and Sequencing

Genomic DNA was extracted following the protocols described by An et al. (2005) with some modifications. DNA extraction was performed using a CTAB-based DNA extraction method. The CTAB lysis buffer contained 2% w/v CTAB (Sigma-Aldrich, Poole, United Kingdom), 100 mM Tris–HCl (pH = 8.0; Fisher Scientific, Fair Lawn, NJ, United States), 20 mM EDTA (pH = 8.0; Fisher), and 1.4 M NaCl (Fisher Scientific). The lysis buffer pH was adjusted to 5.0 prior to sterilization by autoclaving (Doyle and Dickson, 1987). The final DNA was resuspended in 100 μL TE buffer (pH = 8.0; Sigma-Aldrich) and stored at -80°C.

The DNA concentration in each sample was measured using a NanoDrop ND-1000 Spectrophotometer (NanoDrop Technologies, Inc., Wilmington, DE, United States). The integrity of extracted DNA was verified by agarose (1.5%) gel electrophoresis. Subsequently, the V3–V4 region of the 16S rRNA gene was amplified using the following primers: forward 5′-ACT CCT ACG GGR SGC AGC AG-3′ and reverse 5′-GGA CTA CVV GGG TAT CTA ATC-3′ (Xie et al., 2018). The PCRs were performed using the Applied Biosystems Veriti Thermocycler (Thermo Fisher Scientific Co., Ltd., Shanghai, China) in a 20-μL reaction volume. Thermocycling parameters were as follows: initial denaturation at 95°C for 2 min; 30 cycles of further denaturation at 95°C for 15 s, annealing at 50°C for 30 s, and extension at 68°C for 1 min; and a final extension at 68°C for 7 min. All PCRs were performed in triplicate, and products were combined. PCR product integrity was verified by agarose (1.5%) gel electrophoresis, and PCR products were purified with the QIAquick Gel Extraction Kit (Qiagen, Venlo, Netherlands). The concentrations of PCR products were measured using a NanoDrop ND-1000 Spectrophotometer (NanoDrop Technologies, Inc., Wilmington, DE, United States) and subsequently pooled in equal proportions based on DNA concentration. The purified 16S rRNA gene amplicons was sequenced using the paired-end method by Illumina Hiseq 2500 system. The resulting sequences were then screened and filtered for quality and length. Sequences with short reads were extended by merging paired-end reads using FLASH v1.2.7 (Magoc and Salzberg, 2011). Any read pairs that could not be assembled and any single reads were discarded. Sequences were trimmed, quality-filtered and de-convoluted based on the 12 bp barcode sequence. Chimeras were identified and removed using UCHIME v4.2 to obtained effective tags (Edgar, 2010). Subsequently, the sequences were processed and analyzed using Quantitative Insights into Microbial Ecology (QIIME, v1.8.0) as described by Caporaso et al. (2010b). The high-quality sequences were clustered into operational taxonomic units (OTUs) defined by 97% similarity. Taxonomy assignment of representative sequences from each OTU were performed by Ribosomal Database Project classifier (Wang et al., 2007) against its reference database (Cole et al., 2014) with confidence cutoff 0.8. Then representative sequences were aligned against the SILVA bacterial database (SILVA version 128) using PyNAST (Caporaso et al., 2010a). Singletons were removed before further analysis (Bokulich et al., 2013).

### Metagenome Library Preparation and Sequencing

Qualified DNA samples were first cut into smaller fractions by nebulization. Then, using T4 DNA polymerase, the Klenow fragment and T4 polynucleotide kinase convert the fragmentation-produced overhang into blunt ends. After the adenine (A) base was added to the 3′ end of the blunt-ended phosphorylated DNA fragments, the adaptor was ligated to the end of the DNA fragment. Ampure beads were used for purification and elimination the short fragments. PCR amplification were performed to enrich the adapter-ligated DNA fragments. Then, the PCR products were purified with an AxyPrep Mag PCR clean up kit (Axygen, Corning, NY, United States) following the manufacturer’s recommendations. Sample libraries were quantified and analyzed using the Agilent 2100 Bioanalyzer and the ABI StepOnePlus Real-Time PCR system. The qualified libraries were then sequenced on the Illumina HiSeq^TM^ platform.

### Metagenome Assembly and Bioinformatic Analysis

SOAPdenovo2 was used to reassemble high-quality data (Luo et al., 2012). SOAPdenovo results were further assembled with Rabbit to obtain longer contigs (You et al., 2013). For each sample, the reads were assembled in parallel with a series of different k-mer sizes. SOAP2 was used to map the reads back to each assembly result, and selected the optimal k-mer size and assembly results based on contig N50 and mapping rate (Li et al., 2009). Based on the assembly results, MetaGeneMark v2.10 (Tang and Borodovsky, 2010) using default parameters^1^ predicted the presence of open reading frames. Genes from different samples were combined by CD-Hit clustering (Li and Godzik, 2006) (sequence identity threshold, 95%; alignment coverage threshold, 90%).

### CAZyme Annotation and Taxonomic Profiling

The CAZy gene encoding contigs from the metadata were identified and classified based on the CAZymes database (Cantarel et al., 2009)^2^ by the carbohydrate-active enzyme analysis toolkit (CAT) (Park et al., 2010) at an *E*-value of 1 × 10^-5^. Putative plant cell wall polysaccharide-degrading enzymes belonging to different CAZy families were identified and classified based on sequence-based annotation. The CAZyme encoding contigs were analyzed manually for different classes of CAZymes: GHs, GTs, CEs, CBMs, and PLs. Subsequently, the CAZy results obtained were analyzed manually to determine the proportions of the different CAZymes present in the rumen metagenome data.

The CAZy results of the gene were searched against the sequences in the NR database using the BLASTP algorithm with an *E*-value cutoff of 1 × 10^-5^, and the best hits were subjected to analysis with Metagenome Analyzer (MEGAN) (Huson et al., 2011), a program for taxonomic analysis, which could accurately classify DNA sequences as short as 100 bp.

### Pyrosequencing Data Accession Number

The Illumina sequencing raw data for our samples have been deposited in the NCBI Sequence Read Archive (SRA) under accession number: PRJNA522848 (Metagenome) and PRJNA45088 (16S rRNA).

### Statistical Analysis

Community richness and diversity, such as Chao1, and Shannon indices, which are used to illustrate significant differences among samples, were assessed by the program MOTHUR v.1.35.0 (Schloss et al., 2009). The statistical significances were tested by Kruskal–Wallis *H*-test adjusted with false discovery rate, using R (R Core Team, 2016) facilitated with agricolae package (Mendiburu, 2016). The statistical significance was declared at 0.01 < *P*-value < 0.05 “^∗^”, and *P*-value < 0.01 “^∗∗^”. Beta diversity was measured according to Bray–Curtis distances which were calculated by QIIME, and displayed using Principal Coordinate Analysis (PCoA). The significance of grouping in the PCoA plot was tested by analysis of similarity (ANOSIM) in QIIME with 999 permutations (R Core Team, 2016).

The percentage of GH data for each group relative to total GHs identified, enzyme activity, and real-time PCR quantification results were analyzed using the PROC MIXED procedure in SAS 9.4 (SAS Institute Inc.), which included feed, time, and feed × time as the fixed effects, and individual group as the experimental unit.

## Results

### Rumen Metagenome Sequence Data Statistics and Rumen Bacterial Diversity

Metagenome sequencing of the total DNA from 12 rumen samples generated approximately 9.01 gigabases of raw sequence data. The statistical elements of the assemblies were calculated and the metagenomic data analysis statistics are provided in Table 3. At the domain level, evolutionary analysis revealed that ∼95 and ∼90% of sequences were binned to bacteria, ∼0.05 and ∼0.10% to archaea, and ∼0.08 and ∼0.05% to eukaryotes in LF and HF treatments, respectively (Supplementary Table S1). There were 17 bacterial phyla identified in the rumen samples. Among these phyla, *Bacteroidetes*, *Firmicutes*, *Proteobacteria*, *Tenericutes*, and *Verrucomicrobia* were the dominant phyla (Supplementary Table S1), with increasing dietary forage levels, the relative abundance of *Bacteroidetes* and *Verrucomicrobia* increased. As the sampling time increased, the relative abundance of *Bacteroidetes* decreased, whereas that of *Tenericutes* and *Verrucomicrobia* increased.

**Table 3:** Rumen metagenome data assembly analysis statistics using in-house Perl scripts.

Sample	HF		LF	
	BF (0h)^1^	AF (4h)^2^	BF (0h)	AF (4h)
Number of Contigs	1.24 × 10^5^	1.45 × 10^5^	1.26 × 10^5^	1.29 × 10^5^
Assembly Length (bp)	1.67 × 10^8^	1.91 × 10^8^	1.72 × 10^8^	1.77 × 10^8^
N50 (bp)	1,632 ± 53	1,556 ± 76	1,643 ± 95	1,664 ± 147
N90 (bp)	621 ± 7.55	618 ± 5.69	632 ± 4.04	631 ± 11.67
Max Contig (bp)	1.45 × 10^5^	1.48 × 10^5^	1.60 × 10^5^	1.39 × 10^5^
Mix Contig (bp)	500 ± 0.00	500 ± 0.00	500 ± 0.00	500 ± 0.00
Average Size (bp)	1,352 ± 41.79	1,321 ± 33.53	1,365 ± 52.12	1,396 ± 100.24

^1^*BF (0h), before feeding (0 h); ^*2*^AF (4h), after feeding (4 h)*.

**Figure 1:**
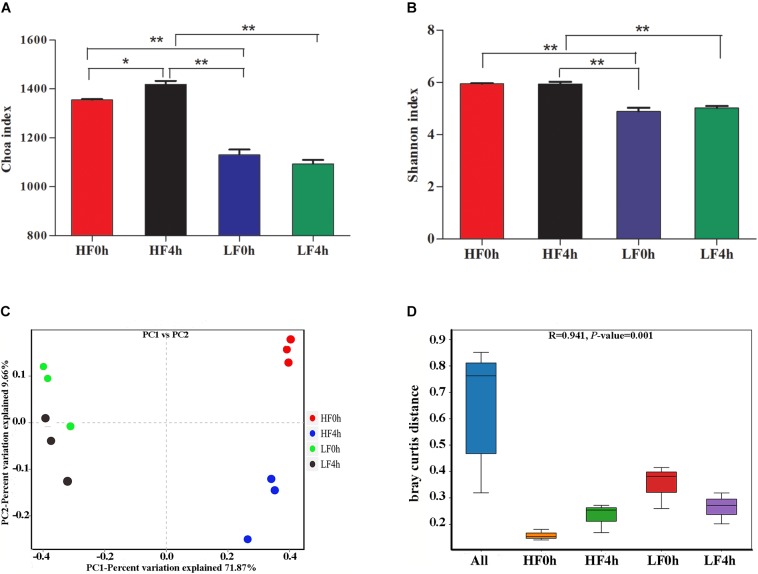
**(A)** Chao 1 indices of alpha diversity base on OUT level. **(B)** Shannon indices of alpha diversity base on OUT level. **(C)** Beta diversity: principal coordinate analysis (PCoA) of bacterial community structure based on Buray–Curtis distances for two treatments of before and after feeding. **(D)** ANOSIM analysis of bacterial community structure based on Buray–Curtis distances for two treatments of before and after feeding. ^∗^(0.01 < *P* < 0.05); ^∗∗^(*P* < 0.01).

In this study, the rumen bacterial alpha diversity was measured by Chao 1 and Shannon indices for different dietary treatments before (0 h) and after (4 h) feeding. The Chao 1 index of HF treatment was significantly (*P* < 0.01) higher compared with that of the LF treatment, and a similar pattern was shown by Shannon indices (Figure 1A,B). Under HF treatment, the Chao 1 index for HF4h was higher than that for HF0h. However, there was no difference in Shannon indices between the two HF groups. This result indicated that forage can increase bacterial richness and diversity. The beta diversities of bacterial communities for different diets before and after feeding were calculated and visualized by PCoA using the Bray–Curtis distance (Figure 1C). The bacterial communities were distinct between HF and LF treatments, and the samples in HF0h and HF4h groups of HF treatment clustered based on different time groups were significant distinction (Figure 1D).

### Rumen Metagenome Mapping for CAZymes and Microbial Composition

A total of 43,630 putative homology-based contigs were inferred with MetaGeneMark and analyzed using the CAZymes analysis toolkit (CAT, see text footnote 2) (Cantarel et al., 2009). CAZymes were determined to belong to different classes (GHs, GTs, CBMs, CEs, and PLs) by CAT are shown in Table 4. GT and CE family abundances (25 and 15 families, respectively) were significantly affected by feed, and GT family abundance was higher under LF treatment than HF treatment (Table 4 and Supplementary Table S2); however, the CE families showed a reverse pattern. The GT families were the second-most abundant in CAZymes; of the 25 GT families identified in this study, enzymes of the GT2 and GT4 families contributed a large proportion (>65%) of the total GTs (Table 4 and Supplementary Table S2). Eight PL families were detected by CAT analysis, and a significant interaction between feed and time was observed for PLs. The abundance of GHs was not affect by diet and time (Table 5), but these families were the most abundant in the rumen metagenomes of CAZymes and included 78 different families (Supplementary Table S2), which accounted for approximately 57% (average of the four groups) of the enzymes categorized in the CAZy database (Table 5). Moreover, 28 CBM families were also detected by CAT analysis.

**Table 4:** Percentage of CAZymes contributed by GH, GT, CBM, CE, and PL (all CAZymes were collectively considered 100%).

	HF		LF		SEM^3^	*P*-value^4^		
	BF	AF	BF	AF		Feed	Time	Feed^∗^Time
	(0h)^1^	(4h)^2^	(0h)	(4h)				
GH	57.47	57.19	56.81	56.16	0.393	NS	NS	NS
GT	18.40	18.55	20.14	20.82	0.386	^∗∗^	NS	NS
CE	11.60	11.74	10.13	10.86	0.037	^∗∗^	NS	NS
PL	2.26	2.35	2.60	2.11	0.007	NS	^∗^	^∗∗^
CBM	10.25	10.16	10.32	10.72	0.052	NS	NS	NS

^1^*BF (0h), before feeding (0 h); ^*2*^AF (4h), after feeding (4 h); ^*3*^SEM for feed × time; ^*4*^NS, not significant (*P* > 0.05); ^∗^, (0.01 < *P* < 0.05); ^∗∗^, (*P* < 0.01). GH stands for glycoside hydrolase, GT for glycosyltransferase, CBM for carbohydrate-binding module, CE for carbohydrate esterase, and PL for polysaccharide lyase*.

**Table 5:** Data for the major GH families identified in our study and other studies.

Enzyme and CAZy family	Major activity	% of each group relative to total GHs identified in each dataset											
		Holstein cow								Holstein	Jersey	Buffalo^c^	HF
		(this study)								cow^a^	cow^b^		cross^d^
		
		HF		LF		SEM^3^	*P*-Value^4^			AF	AF	AF	AF
										(1h)	(no found)	(3h)	(2h)
		BF	AF	BF	AF		Feed	Time	Feed^∗^time				
		(0h)^1^	(4h)^2^	(0h)	(4h)								
**Cellulases**					
GH5	Cellulases, endoglucanase	4.22	3.89	3.52	3.68	0.008	^∗∗^	NS	^∗^	9.41	7.45	1.51	2.40
GH6	endoglucanase	0.00	0.00	0	0					0.35	0.31	0.00	0.00
GH7	endoglucanase	0.00	0.00	0	0					0.00	0.00	0.00	0.00
GH9	endoglucanase	1.54	1.39	0.93	0.94	0.003	^∗∗^	NS	NS	13.71	2.48	0.26	0.80
GH44	endoglucanase	0.00	0.00	0	0					0.35	0.00	0.00	0.03
GH45	endoglucanase	0.00	0.00	0	0					3.94	0.00	0.05	0.08
GH48	cellobiohydrolases	0.00	0.00	0	0					7.65	0.00	0.00	0.03
GH88	β-glucuronyl hydrolase	0.32	0.26	0.18	0.18	0.001	^∗∗^	NS	NS	0.00	0.00	1.61	0.00
GH95	α-L -fucosidase	2.31	2.40	3.46	2.36	0.004	NS	NS	NS	0.00	0.00	0.00	0.00
Subtotal		8.39	7.94	8.09	7.16					35.40	10.25	3.43	3.34
**Endo-hemicellulases**					
GH8	Endoxylanses,	0.50	0.62	0.82	0.79	0.003	^∗∗^	NS	NS	1.11	0.00	0.00	0.22
GH10	endo-1,4-β-xylanases	0.80	0.95	0.94	0.89	0.003	NS	NS	^∗^	8.48	10.87	2.65	1.30
GH11	xylanases	0.00	0.00	0	0					4.78	0.00	0.16	0.20
GH12	xyloglucanases	0.00	0.00	0	0					0.00	0.31	0.00	0.62
GH26	β-mannanase and xylanases	1.01	1.00	1.01	0.92	0.002	NS	NS	NS	3.12	0.31	1.04	0.3
GH28	Polygalacturonase	3.31	3.33	4.23	4.1	0.007	^∗∗^	NS	NS	1.26	0.00	0.21	0.2
GH53	endo-1,4-β-galactanases	1.24	1.38	1.4	1.41	0.005	^∗^	NS	NS	1.84	5.59	1.46	6.75
Subtotal		6.86	7.28	8.41	8.11					20.59	17.08	5.51	10.07
**Oligosaccharide-degrading enzymes**					
GH1	β-glucosidases	0.04	0.04	0.09	0.07		^∗^	NS	NS	5.07	3.11	0.05	3.75
GH2	β-galactosidases	9.53	9.72	9.16	8.88	0.043	^∗∗^	NS	NS	6.01	4.97	10.03	5.48
GH3	β-glucosidases	10.82	11.20	12.51	12.4	0.035	^∗∗^	NS	NS	12.20	14.91	18.35	9.37
GH13	α-Amylase	5.14	5.28	6.76	2.22	0.050	^∗∗^	NS	NS	0.00	4.66	3.95	5.48
GH18	Chitinase	0.41	0.38	0.63	0.59	0.001	^∗∗^	NS	NS	0.00	0.62	0.42	5.06
GH20	β-Hexosaminidase	1.90	1.70	0.74	0.8	0.007	^∗∗^	NS	NS	0.00	0.31	3.17	2.75
GH27	α-Galactosidase	0.71	0.65	0.32	0.31	0.001	^∗∗^	NS	NS	0.00	0.31	1.77	0.40
GH29	α-L -fucosidosis	1.90	1.65	1.15	1.21	0.006	^∗∗^	NS	^∗^	1.64	0.93	2.29	0.82
GH31	α-Glucosidase	4.01	4.18	4.38	4.3	0.023	NS	NS	NS	0.00	1.55	5.09	2.00
GH32	Invertase, endo-inulinase	1.76	1.91	2.36	2.31	0.005	^∗∗^	NS	NS	0.00	2.48	1.56	2.25
GH35	β-galactosidases	0.76	0.77	0.94	0.92		^∗∗^	NS	NS	0.67	0.62	0.78	0.48
GH38	α-mannosidases	0.03	0.02	0.01	0.01		^∗∗^	^∗∗^	^∗∗^	0.47	0.31	0.26	1.89
GH39	β-xylosidases	0.01	0.01	0.02	0.01		NS	NS	^∗∗^	0.37	5.59	0.00	4.10
GH42	β-galactosidases	0.04	0.03	0.08	0.05		^∗∗^	^∗^	^∗^	0.79	0.00	0.10	0.04
GH43	arabino/xylosidases	10.24	10.63	11.29	11.34	0.056	^∗∗^	NS	NS	9.35	8.70	2.96	2.58
GH52	β-xylosidases	0.00	0.00	0	0					0.00		0.00	
GH57	α-Amylase	1.23	1.20	1.04	1.05	0.001	^∗∗^	NS	NS	0.00	0.00	0.57	0.60
GH92	α-1,2-mannosidase	3.29	2.83	1.98	2.3	0.018	^∗∗^	NS	^∗∗^		1.24	7.02	2.58
GH94	cellobiose phosphorylase	0.91	0.94	1.15	0.97	0.001	^∗∗^	^∗^	^∗∗^	33.43	0.00	1.14	
GH97	α-Glucosidase	4.87	5.00	5.06	5.13	0.008	NS	NS	NS	0.00	0.00	5.98	1.28
GH130	β-1,4-Mannosylglucose phosphorylase	1.13	1.11	0.97	0.94	0.001	^∗∗^	NS	NS	0.00	0.00	0.00	0.69
Subtotal		58.73	59.25	60.64	55.81					40.19	50.31	65.49	51.59
**Debranching enzymes**					
GH23	Peptidoglycan lyase	1.73	1.64	1.35	1.51	0.003	^∗∗^	NS	^∗^	0.00	1.55	3.17	4.99
GH33	*trans*-Sialidase	1.06	0.89	0.42	0.45	0.002	^∗∗^	NS	^∗^	0.00	1.24	0.31	1.42
GH51	α-L -arabino furanosidases	3.61	3.65	3.88	3.67	0.012	NS	NS	NS	1.50	0.31	1.35	0.60
GH54	α-L -arabino furanosidases	0.23	0.21	0.013	0.007		^∗∗^	NS	^∗^	0.08	0.00	0.42	0.13
GH62	α-L -arabino furanosidases	0.00	0.00	0	0					0.00	0.00	0.00	0.00
GH67	α-glucuronidases	0.88	0.94	1.11	1.14	0.001	^∗∗^	NS	NS	0.43	0.00	2.49	0.36
GH77	4-α-Glucanotransferase	0.37	0.33	0.61	0.65		^∗∗^	NS	^∗^		0.31	2.81	1.61
GH78	α-L -rhamnosidase	2.19	2.03	1.47	1.37	0.005	^∗∗^	^∗^	NS	1.80	4.04	2.86	1.73
GH84	N-Acetyl	0.01	0.01	0.14	0.24		^∗∗^	^∗∗^	^∗∗^	3.81	0.31	0.21	2.06
GH103	β-glucosaminidase transglycosylase	0.00	0.00	0.27	0.32	0.001	^∗∗^	NS	NS	0.00	0.00	0.00	1.36
GH127	α-Galactosidase	1.38	1.34	1.29	1.26	0.002	^∗^	NS	NS	0.00	0.00	4.26	1.57
Subtotal		11.46	11.04	10.55	10.62					7.62	7.76	17.88	15.84

^1^*BF (0h), before feeding (0 h); ^*2*^AF (4h), after feeding (4 h); ^*3*^SEM for feed × time; ^*4*^NS, not significant (*P* > 0.05); ^∗^, (0.01 < *P* < 0.05); ^∗∗^, (*P* < 0.01). (a) Dai et al. (2015), shotgun metagenomic sequencing using the Roche 454 GS FLX. Each number is the average of two Holstein cow rumen samples. (b) Wang et al. (2013), shotgun metagenomic sequencing using the Roche GS FLX. Each number is the average of two Jersey cow rumen samples. (c) Patel et al. (2014), shotgun metagenomic sequencing using Life Ion Torrent PGM. Each number is the average of four Buffalo rumen samples. (d) Jose et al. (2017b), shotgun metagenomic sequencing using Illumina MiSeq. Each number is the average of three HF cross rumen samples*.

**Figure 2:**
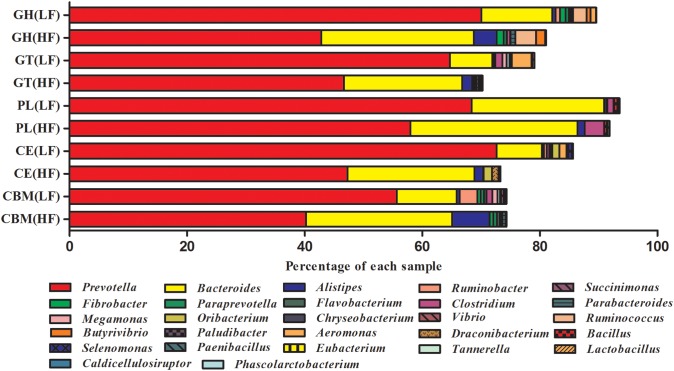
Percent contributions of CAZymes from the major microbial communities in cattle rumens. Each graph shows the abundance of 15 genera that are the major contributors of CAZymes to the Holstein cow rumen ecosystem. GH stands for glycoside hydrolase, GT for glycosyltransferase, CBM for carbohydrate-binding module, CE for carbohydrate esterases, and PL for polysaccharide lyase. All genera in each sample totaled 100%.

Phylogenetic analysis of CAZyme contigs showed that *Prevotella* and *Bacteroides* primarily contributed CAZyme-encoding gene fragments of the GH, GT, CBM, CE, and PL families in the Holstein cow rumen metagenome (Figure 2). The number of enzymes that belonged to *Prevotella* was significantly higher in LF than HF groups; the reverse was observed for *Bacteroides*. *Alistipes* was found in all five categories, with its highest abundance in the CBM family of the HF groups, followed by the GH, GT, PL, and CE families of the HF groups (Figure 2). The number of enzymes from *Ruminococcus*, one of the most dominant cellulolytic bacteria of the GH families, was higher under HF treatment than LF treatment (Figure 2). *Butyrivibrio* and *Fibrobacter* were detected in the GH families, and were higher under HF treatment than LF treatment (Figure 2). *Prevotella*, *Bacteroides*, *Alistipes*, *Clostridium*, and *Ruminococcus* were found in all five CAZyme categories and were the primary contributors of CAZymes.

### Comparison of GH Families of Cows Fed Two Different Diets

Out of the 78 GH families identified, 39 were reported to be involved in the direct degradation of plant fiber (Table 5). Four GH families (GH5, GH9, GH88, and GH95) were mainly found to be associated with cellulolytic functions, and represented 7.96 and 8.53% (average of BF0h and AF4h) of the total GHs under HF and LF treatments, respectively. The abundances of GH5, GH9, and GH88 were significantly affected by diets, and GH5 was also affected by feed and time interaction.

Five GH families (GH8, GH10, GH26, GH28, and GH53) were detected in our study, and have important roles in hemicellulose degradation (Table 5). Time treatment did not affect GH8, GH10, GH26, GH28, and GH53, but GH8 and GH28 were significantly (*P* < 0.01) affected by feed. Percentage of GH10 was also significantly affected (*P* < 0.01) by interaction between feed and time. The pivotal series of enzymes that are responsible for hydrolysis of the main chain of galactooligosaccharides, such as galacturonases and endo-1,4-galactanase, were present in the GH28 and GH53 families. These enzymes represented approximately 66% (average of the four groups) of the endo-hemicellulases, which were more abundant under LF treatment; however, there was no difference between the LF0h and LF4h groups.

Oligosaccharide-degrading enzymes had a greater abundance of GH families than other cellulose-degrading enzymes, and represented 60.64, 55.81, 58.73, and 59.25% of the total GHs in the LF0h, LF4h, HF0h, and HF4h groups, respectively (Table 4). No noticeable differences of the GH31, GH39, and GH97 families were observed between HF and LF treatments; however, the other GH families of oligosaccharide-degrading enzymes were affected by feed (Table 5). Among them, GH38, GH42, and GH94 were also affected by time and interaction between feed and time. Among the GHs responsible for oligosaccharide degradation (oligo-GHs), GH2, GH3, GH13, GH31, and GH43 were the main GHs, which accounted for ∼70% of all oligo-GHs (Table 5). Of those GHs, GH3 was the most abundant (20.63, 22.22, 18.42, and 18.90% of total oligo-GHs in the LF0h, LF4h, HF0h, and HF4h groups, respectively). The second most abundant was the coding endoglucanase GH43, and the abundance was significantly (*P* < 0.01) higher under LF treatment than HF treatment.

Debranching enzymes were also identified by CAT analysis and belonged to the GH23, GH33, GH51, GH54, GH67, GH78, and GH127 families; of these, GH51, GH67, and GH78 were more abundant and have the function of α-L-arabinofuranosidases, α-glucuronidases, and α-L-rhamnosidases, respectively (Table 5). GH51 was the most abundant, but was not affected by feed, time, or the interaction between feed and time. GH78 was significantly (*P* < 0.01) more abundant under HF treatment than LF treatment.

### Enzyme Activity of Cows Fed Two Different Diets

Endo-1,4-glucanase (the CMCase) and β-glucosidase were the main cellulases. In this study, the CMCase was affected by time, and at 0 h before feeding had significantly higher (*P* < 0.01) activity than at 4 h after feeding (Table 6). HF-fed cows had greater β-glucosidase activity than LF-fed cows. β-glucosidase activity at 4 h after feeding was significantly (*P* < 0.01) higher than that at 0 h before feeding, and β-glucosidase activity was also affected by a significant (*P* < 0.01) interaction between feed and time (Table 6). Xylanase and β-xylosidase are the main hemicelluloses. Xylanase activity in HF-fed cows was significantly (*P* < 0.01) higher than that in LF-fed cows, and at 4 h after feeding was significantly (*P* < 0.01) decreased compared with at 0 h before feeding. Dietary treatment did not affect β-xylosidase activity, but substantial variation was observed between the treatments. For example, β-xylosidase activity ranged from, on average, 3.49 U in HF-fed cows to 3.04 U in LF-fed cows.

**Table 6:** Enzyme activity of cows fed two different diets.

	HF		LF		SEM^3^	*P*-value^4^		
	BF	AF	BF	AF		Feed	Time	Feed^∗^time
	(0h)^1^	(4h)^2^	(0h)	(4h)				
CMCase	8.94	7.99	9.51	7.51	0.317	NS	^∗∗^	NS
β-glucosidase	15.75	18.03	16.61	21.97	0.382	^∗∗^	^∗∗^	^∗∗^
Xylanase	13.16	10.81	11.62	9.18	0.758	^∗^	^∗∗^	NS
β-xylosidase	3.54	3.44	2.91	3.18	0.114	NS	NS	NS

^1^*BF (0h), before feeding (0 h); ^*2*^AF (4h), after feeding (4 h); ^*3*^SEM for feed × time; ^*4*^NS, not significant (*P* > 0.05); ^∗^, (0.01 < *P* < 0.05); ^∗∗^, (*P* < 0.01)*.

### Fiber-Degrading Bacteria Characterization and Predominant Fiber-Degrading Bacteria Relative Quantification

After statistical analysis, all genus-level data were converted back into CAZyme (GH) percent relative abundance are presented in Table 7. The cel-GH (GH families responsible for cellulose degradation) reads mainly originated from *Bacteroides* and *Prevotella*, and were also affected by feed. Our data showed that *Fibrobacter* content was the highest, and these bacteria play an important role in cellulose degradation. *Butyrivibrio* relative abundance was affected by time, and was higher at 4 h after feeding than at 0 h before feeding.

**Table 7:** Taxonomic affiliation of putative cellulase, hemicelluase, and oligosaccharide-degrading enzymes.

	HF		LF		SEM^3^	*P*-value^4^		
	BF	AF	BF	AF		Feed	Time	Feed^∗^time
	(0h)^1^	(4h)^2^	(0h)	(4h)				
**Cellulases (genera)**					
*Alistipes*	0.026	0.017	0.003	0.003		^∗∗^	NS	NS
*Bacteroides*	1.949	1.801	0.105	0.944	0.0032	^∗∗^	^∗^	NS
*Prevotella*	3.411	3.881	5.923	5.716	0.0389	^∗^	NS	NS
*Ruminococcus*	0.014	0.009	0.007	0.009		NS	NS	NS
*Butyrivibrio*	0.468	0.621	0.521	0.767	0.0037	NS	^∗^	NS
*Fibrobacter*	0.279	0.417	0.251	0.240	0.0036	NS	NS	NS
*Paraprevotella*	0.026	0.023	0.007	0.006		^∗∗^	NS	NS
*Parabacteroides*	0.032	0.021	0.003	0.001		^∗∗^	^∗^	^∗^
**Hemicellulases (genera)**					
*Alistipes*	0.130	0.119	0.035	0.036		^∗∗^	NS	NS
*Bacteroides*	1.593	1.514	0.812	0.839	0.0025	^∗∗^	NS	NS
*Butyrivibrio*	0.074	0.025	0.204	0.055	0.0014	^∗^	^∗^	NS
*Prevotella*	3.532	4.071	6.278	6.011	0.0270	^∗∗^	NS	^∗^
*Ruminococcus*	1.364	1.118	0.736	0.826	0.0137	^∗∗^	NS	NS
*Fibrobacter*	0.193	0.561	0.179	0.175	0.0073	^∗^	^∗^	^∗^
*Paraprevotella*	0.008	0.004	0.017	0.027		^∗∗^	NS	^∗^
**Oligosaccharide-degrading enzymes**					
*Alistipes*	2.013	1.825	0.266	0.275	0.0073	^∗∗^	NS	NS
*Bacteroides*	11.847	11.021	5.848	5.682	0.1127	^∗∗^	NS	NS
*Prevotella*	23.322	26.878	38.880	39.036	3.0056	^∗∗^	NS	NS
*Butyrivibrio*	0.090	0.147	0.061	0.042	0.0015	^∗∗^	NS	^∗^
*Draconibacterium*	0.252	0.165	0.000	0.000	0.0022	^∗∗^	NS	NS
*Fibrobacter*	0.022	0.017	0.000	0.000		^∗∗^	NS	NS
*Ruminococcus*	0.028	0.026	0.038	0.024		NS	NS	NS
*Clostridium*	0.019	0.019	0.052	0.046	0.0018	NS	NS	NS
*Paludibacter*	0.319	0.260	0.008	0.012		^∗∗^	^∗^	^∗^
*Roseburia*	0.000	0.000	0.288	0.014	0.0024	^∗^	^∗^	^∗^
**Main fiber-degrading bacterial relative expression (%) of total bacterial**					
*Ruminococcus flavefaciens*	1.972	2.659	2.332	2.638	0.0421	NS	^∗^	NS
*Ruminococcus albus*	0.161	0.309	0.357	0.247	0.0021	NS	NS	^∗∗^
*Fibrobacter succinogenes*	0.186	0.357	0.322	0.518	0.0017	^∗∗^	^∗∗^	NS
*Butyrivibrio fibrisolvens*	0.562	0.667	0.388	0.180	0.0001	^∗∗^	NS	^∗∗^

^1^*BF (0h), before feeding (0 h); ^*2*^AF (4h), after feeding (4 h); ^*3*^SEM for feed × time; ^*4*^NS, not significant (*P* > 0.05); ^∗^(0.01 < *P* < 0.05); ^∗∗^(*P* < 0.01)*.

The majority of the hemi-GH (GH families responsible for hemicellulose degradation) reads in samples originated from *Bacteroides*, *Prevotella*, and *Ruminococcus* (Table 7), which were significantly affected by feed, and *Bacteroides* and *Prevotella* abundances were higher under LF treatment than under HF treatment. Under HF treatment, *Fibrobacter* relative abundance was higher than that under LF treatment, and after feeding was higher compared with before feeding; there was a significant (*P* < 0.05) interaction between feed and time related to *Fibrobacter* relative abundance.

The oligo-GHs (GHs responsible for oligosaccharide degradation) contained the most bacterial genera and the greatest relative abundance compared with cel-GHs and hemi-GHs (Table 7). The bacterial genera were primarily *Bacteroides* and *Prevotella*, and the relative abundances were significantly (*P* < 0.01) higher compared with that of cel-GHs and hemi-GHs. *Bacteroides* and *Prevotella* were the main contributors to oligosaccharide degradation, followed by *Alistipes*, *Draconibacterium*, *Paludibacter*, and *Clostridium*. Based on our data, the relative abundances of *Butyrivibrio*, *Fibrobacter*, and *Ruminococcus*, which were the major producers of cel-GH, appeared to contribute little to oligo-GH hydrolysis.

Abundance of the predominant fiber-degrading bacteria was measured by Real-Time qPCR quantification in two treatments, both before or after feeding. *Ruminococcus flavefaciens* and *R. albus* bacteria were derived from *Ruminococcus.* Based on cDNA level, the *R. flavefaciens* activity at 4 h after feeding was significantly higher compared with that at 0 h before feeding. *Fibrobacter succinogenes* activity was significantly affected by feed and time. *Butyrivibrio fibrisolvens* activity was significantly (*P* < 0.01) affected by feed, and a significant (*P* < 0.01) interaction between feed and time was observed for *R. albus* and *B. fibrisolvens* activity.

## Discussion

The rumen is a complex ecosystem that harbors a wide variety of microorganisms. *Bacteroidetes*, *Firmicutes*, and *Proteobacteria* were the most predominant bacteria in the rumen, which was recognized by most previous studies (Dai et al., 2015; Wang et al., 2017; Zhang et al., 2017). Martinezgarcia et al. (2012) showed that *Verrucomicrobia* could hydrolyze diverse polysaccharides. In the present study, *Verrucomicrobia* was significantly higher in the HF group than in the LF group, which might indicate that *Verrucomicrobia* plays an important role in the degradation of plant cell wall polysaccharides.

Linking phylogeny to function is a recurrent question in microbiology of rumen. A growing number of rumen metagenomic studies have shown physical and chemical properties of functional genes in rumen samples, which provided a way to evaluate the relationship of phylogeny to function (Hess et al., 2011; Singh et al., 2015). Therefore, analysis of CAZyme-regulating gene abundance and categories in cow rumens could help characterize fiber degradation. Metagenomic analysis of microbial consortia enriched in the rumen showed that GH families were most abundant.

### Rumen Metagenome Mapping for CAZymes

The GHs comprise a large group of enzymes involved in the metabolism of polysaccharides such as starch, cellulose, xylan, and chitin (Stewart et al., 2018). Because of the most abundance and wide distribution of GH-encoding genes across genomes, this enzyme class is the best characterized of the CAZymes (Berlemont and Martiny, 2015). GTs were the second most abundant CAZy family in the Holstein cow rumen, and were reported to catalyze the activated oligosaccharides or glycosidic bonds to different receptors (e.g., proteins, nucleic acids, oligosaccharides, lipids, and small molecules) (Lairson et al., 2008). The results of this study showed that the GTs family abundance was higher under LF treatment and was significantly affected by feed. 25 GT families were identified in the rumen metagenome, and GT2 and GT4 family enzymes were a large proportion (>65%) of the total GTs (Supplementary Table S2); these results were similar to those for Indian crossbred cattle (Jose et al., 2017b). The results showed that the CE families was higher in HF treatment than that in LF treatment. The CE1 family made up 36.30 and 30.60% of all CE families in the rumen of HF- and LF-fed cows, respectively. These results indicate that the greater abundance of CE families under HF treatment was caused by the presence of the CE1 family. Feruloyl esterase, which is encoded by the CE1 family, is essential for plant fiber degradation and is the predominant member of the CE family (Biely, 2012). PLs, which cleave glycosidic bonds in acidic polysaccharides (Stewart et al., 2018), were the least abundant CAZyme classes in the cow rumen metagenome, and this result was consistent with that of Jose et al. (2017a).

### GH Family Composition and Diversity

The GH families include enzymes that hydrolyze glycosidic bonds by various glucosides or oligosaccharides, and was the most abundant family among those included in the CAZy databases (Solden et al., 2018). Among the 156 GH families in the CAZy database^3^, 78 families were presence in the Holstein cow rumen metagenome in this study, which indicates that the Holstein cow rumen might undergo an intricate process to break down plant cell wall polysaccharides. Our results revealed that genes that encoded cellulases (cel-GH) mainly belonged to the GH5, GH9, GH88, and GH95 families, which was also determined by previous studies (Wang et al., 2013; Patel et al., 2014; Solden et al., 2018). Among cel-GHs, the GH5 family was the most abundant, and GH5 is well known with activities of β-1,4-endoglucanase and β-1,4-endomannanase, and GH9 also have endoglucanase activity (Maharjan et al., 2018). Naas et al. (2014) predicted that the GH5 and GH9 families could break down cellulose into cellobiose and act on β-(1,4)-linked glucose units in amorphous cellulose and β-glucan. In this study, endoglucanase (CMCase) activity was higher under LF treatment than HF treatment (Table 6), and was similar to GH5 and GH9 relative abundances.

There are five GH families that are degrading hemicellulose and belong to hemicellulase, referred to as hemi-GHs. Among hemi-GH, GH28 family was the most abundant, followed by GH53, and the relative abundant of both under LF treatment was higher than under HF treatment. Polygalacturonase were dominated belong to GH28 and acting α-1,4-glycosidic bond, which plays an important role in pectin digestion (Zhao et al., 2014). GH8 representing the endoxylanses has been found to be the most critical hydrolase and in xylan hydrolase system and it hydrolyzes xylan into oligosaccharides and xylodisaccharides. Then β-xylosidase hydrolyzes oligosaccharides and xylodisaccharides into xylose. In present study, the content of xylose in HF treatment was higher than in LF treatment (Table 6, β-xylosidase). Our results indicate that xylose has feedback inhibition on endoxylanase. Putative arabinogalactan end-1,4-β-galactosidase gene were found belong to GH53, and degrading the hemicellulose side chains and pectin. The large number of CAZy genes found is indicative of the potential of various rumen bacteria to utilize carbohydrates as their main substrates (Supplementary Table S3).

Patel et al. (2014) reported that oligosaccharide-degrading enzymes in buffalo were more abundant by approximately 64%, with GH43 being the most abundant family, followed by GH3. The GH3 family encodes β-glucosidase, xylan, β-1,4-xylosidase, and glucosylceramidase. β-glucosidase play an central role both in the production of glucose and, crucially, in the alleviation of the product inhibition of cellobiose during the cellulose degradation process itself (Agirre et al., 2016). In this study, among the four groups, β-glucosidase was the highest in the LF 4h group, this indicates that the LF 4h group has more glucose, then, glucose is uptaken by rumen microbes and rapidly degraded into volatile fatty acids by enzymes, which provides energy for the host and have lowest pH (Kuruti et al., 2017). The function of GH3 indicates that it is a rate-limiting enzyme in the rumen cellulose degradation process.

Debranching enzymes, such as β-xylosidase, α-larabinofuranosidase, and arabinanase, are crucial components of hemicellulolytic enzyme that promote endo-enzymes acting on their substrate (Comtetmarre et al., 2017). GH51 (α-l-arabinofuranosidases), GH67 (α-glucuronidases), and GH78 (α-L-rhamnosidase) were the main debranching enzymes (Patel et al., 2014; Dai et al., 2015; Jose et al., 2017a). In our study, GH51 (α-L-arabinofuranosidases/endoglucanase) was the most abundant debranching enzyme, which indicated that was the main debranching enzyme.

### Microbial Community Analysis of Putative CAZyme Contigs and Cellulase Degradation

In this report, we present a metagenomic analysis of the fiber-degrading microbiomes of cows fed two different diets. Previous studies showed that rumen microbiota play vital roles in plant fiber degradation (Kittelmann and Janssen, 2011; Li et al., 2012). At the genus level, the comparative abundances of the 15 most abundant genera among GHs, GTs, CEs, PLs, and CBMs are shown in Figure 2 and indicate that *Prevotella* and *Bacteroides* contribute a significant proportion of CAZymes under LF treatment, which is consistent with the findings of Stewart et al. (2018). *Prevotella* and *Bacteroides* both belong to the phylum *Bacteroidetes*, and Solden et al. (2018) reported that *Bacteroidetes* play an important role in rumen carbon degradation, and the abundance of *Bacteroides* increased with the increase of fiber in diets (Pitta et al., 2014). The results of this study showed that the *Prevotella* abundance was highest in GHs, GTs, CEs, PLs, and CBMs, and significantly higher in LF treatment than HF treatment, which was consistent with the findings of previous studies (Bekele et al., 2010), and indicated that they might be the essential microorganisms and maintained normal digestive function of the rumen. *Alistipes* in HF groups was more abundant in CAZyme class GHs and CBMs than in LF groups. He et al. (2015) showed that a reduction in the amount of *Alistipes* and *Bacteroides* is known to be associated with low-carbohydrate diets.

In the rumen, *Bacteroides*, *Butyrivibrio*, *Ruminococcus*, and *Fibrobacter* were the dominant fibrolytic microorganisms. The enzymes produced by these microbial communities are reported to have the potential to digest plant polymers, such as cellulose, hemicellulose, and oligosaccharide-degrading enzymes (Whitman, 2015; Díaz et al., 2017). In present study, the results of *Bacteroides* relative abundances further confirmed that *Bacteroides* was the main producer of oligosaccharide-degrading enzymes. Meanwhile, *Ruminococcus* relative abundance in hemicellulases was higher under HF treatment than LF treatment, which indicates that *Ruminococcus* may play an important role in hemicellulose degradation. *Prevotella* abundance was relatively highest in oligosaccharide-degrading enzymes (Table 7). This could be explained by the fact that *Prevotella* can degrade and utilize starch and plant cell wall polysaccharides, such as xylan and pectin, but cannot degrade cellulose (Zened et al., 2013). *Alistipes* was higher in oligosaccharide-degrading enzymes and significantly affected by feed. The results of this study indicated that *Alistipes* are associated with carbohydrate, especially oligosaccharide metabolism, as previously reported (Wang et al., 2017). We also found that *Draconibacterium* and *Paludibacter* were associated with oligosaccharide-degrading enzymes, and with high relative abundance (Supplementary Table S3). *Paludibacter* is involved in oligosaccharide degradation in plants (Ghanbari et al., 2016). *Draconibacterium*, which belongs to phylum *Bacteroidetes*, encodes a variety of enzymes and proteins required for glycolysis, the Krebs cycle, the pentose phosphate pathway, and oxidative phosphorylation, which reflects the integrity of the *Draconibacterium* metabolic pathway (Li et al., 2016), which could explain the *Draconibacterium* was the main producer of oligosaccharide-degrading enzymes. Furfure work should investigate the glycan-degrading abilities of these different bacteria to determine if the bacteria evolved to specialize on different diets.

## Conclusion

In conclusion, this study investigated individual changes in CAZymes, and microbial composition variation in response to the change of diet and time. We revealed that dietary treatment has significant effects on the CAZymes in cattle rumens. The dominant phyla and genera composition of the CAZymes varied among the four groups, the *Bacteroides*, *Fibrobacter*, and *Ruminococcus* largely increased as forage increased, and were identified as the key contributors of CAZymes, which indicated that the disparity amongst these two factors should be taken into account when exploring the CAZymes and related microbial composition. Therefore, this study can enhance our understanding of a biomass conversion system and demonstrates that numerous enzymes are involved in cellulose degradation in the cow rumen, which contributes to the improvement of forage utilization in ruminant nutrition.

## Ethics Statement

All animal studies were conducted according to the animal care and use guidelines of the Animal Care and Use Committee of Animal Science and Technology College, Northeast Agricultural University.

## Author Contributions

LW and YZ designed the research. LW conducted the research. GZ and HoX analyzed the data. LW and HaX wrote the manuscript. All authors approved the final manuscript.

## Conflict of Interest Statement

The authors declare that the research was conducted in the absence of any commercial or financial relationships that could be construed as a potential conflict of interest.

## Footnotes

1 http://exon.gatech.edu/GeneMark/

2 http://www.cazy.org

3 http://www.cazy.org/Glycoside-Hydrolases.html
