# Supplementary material for: Metagenomic Analyses of Microbial and Carbohydrate-Active Enzymes in the Rumen of Holstein Cows Fed Different Forage-to-Concentrate Ratios
Source: Front Microbiol. 2019 Mar 29;10:649. doi: 10.3389/fmicb.2019.00649 (PMC6449447; doi:10.3389/fmicb.2019.00649)
Supplement: Supplementary file 1 [file Table_1.docx]

Metagenomic Analyses of Microbial and Carbohydrate-Active Enzymes in the Rumen of Holstein Cows Fed Different Forage-to-Concentrate Ratios

Lijun Wang, Guangning Zhang, Hongjian Xu, Hangshu Xin* and Yonggen Zhang*

College of Animal Science and Technology, Northeast Agricultural University, Harbin, China

^*^The corresponding author: Yonggen Zhang and Hangshu Xin

Tel) +86-451-55190840, Fax) +86-451-55190840

E-mail: [zhangyonggen@sina.com](mailto:zhangyonggen@sina.com); [xinhangshu@163.com](mailto:xinhangshu@163.com)

**Supplement Table 1.**

**The relative abundance (%) of bacterial phyla of cows fed two different diets**

|  | HF | | LF | | SEM^3^ | *P*-value^4^ | | |
| --- | --- | --- | --- | --- | --- | --- | --- | --- |
|  | BF(0h)^1^ | AF(4h)^2^ | BF(0h) | AF(4h) |  | Feed | Time | Feed*time |
| Domain |  |  |  |  |  |  |  |  |
| Bacteria | 88.951 | 90.787 | 95.532 | 95.656 | 0.6482 | ** | NS | NS |
| Eukaryota | 0.0483 | 0.0500 | 0.0703 | 0.0867 | 0.0001 | ** | NS | NS |
| Viruses | 0.1667 | 0.1651 | 0.6133 | 0.8917 | 0.0428 | ** | NS | NS |
| Archaea | 0.1063 | 0.1010 | 0.0403 | 0.0522 | 0.0000 | ** | NS | * |
| Unclassfied | 10.728 | 8.8964 | 3.7443 | 3.3135 | 0.6706 | ** | NS | NS |
| Phylum |  |  |  |  |  |  |  |  |
| *Actinobacteria* | 0.1416 | 0.1140 | 0.0409 | 0.1987 | 0.0016 | NS | NS | * |
| *Armatimonadetes* | 0.0101 | 0.0229 | 0.0000 | 0.0011 | 0.0000 | * | NS | NS |
| *Bacteroidetes* | 69.248 | 54.117 | 26.570 | 24.145 | 7.4910 | ** | ** | * |
| *Cyanobacteria* | 0.1394 | 0.1165 | 0.4824 | 0.2835 | 0.0000 | ** | ** | ** |
| *Elusimicrobia* | 0.0328 | 0.0368 | 0.0024 | 0.0006 | 0.0000 | ** | NS | NS |
| *Fibrobacteres* | 0.1101 | 0.0413 | 0.0663 | 0.0024 | 0.0013 | NS | * | NS |
| *Firmicutes* | 25.882 | 36.094 | 57.954 | 57.059 | 17.171 | ** | NS | NS |
| *Gracilibacteria* | 0.0016 | 0.0031 | 0.0085 | 0.0030 | 0.0000 | NS | NS | NS |
| *Lentisphaerae* | 0.2475 | 0.2952 | 0.0000 | 0.0006 | 0.0043 | ** | NS | NS |
| *Planctomycetes* | 0.00155 | 0.0076 | 0.0000 | 0.0000 | 0.0000 | ** | ** | ** |
| *Proteobacteria* | 0.780.3 | 1.3699 | 12.591 | 14.501 | 0.9983 | ** | NS | NS |
| *SR1_(Absconditabacteria)* | 0.2292 | 0.2349 | 0.6563 | 0.2655 | 0.0040 | ** | ** | ** |
| *Saccharibacteria* | 0.5173 | 1.6227 | 0.4238 | 1.1283 | 0.0711 | NS | ** | NS |
| *Spirochaetae* | 0.1815 | 0.1673 | 0.0415 | 0.0095 | 0.0011 | ** | NS | NS |
| *Synergistetes* | 0.0537 | 0.09909 | 0.0913 | 0.1214 | 0.0002 | * | * | NS |
| *Tenericutes* | 1.1177 | 2.1416 | 0.7622 | 1.8117 | 0.0616 | NS | ** | NS |
| *Verrucomicrobia* | 1.3742 | 3.4543 | 0.3248 | 0.4697 | 0.1102 | ** | ** | ** |

^1^ BF(0h), before feeding (0 h); ^2^ AF(4h), after feeding (4 h); ^3^ SEM for feed × time; ^4^ NS, not significant (*P* > 0.05);

*, (0.01 < *P* < 0.05); **, (*P* < 0.01)
