# Supplementary material for: Metagenomic Analyses of Microbial and Carbohydrate-Active Enzymes in the Rumen of Holstein Cows Fed Different Forage-to-Concentrate Ratios
Source: Front Microbiol. 2019 Mar 29;10:649. doi: 10.3389/fmicb.2019.00649 (PMC6449447; doi:10.3389/fmicb.2019.00649)
Supplement: Supplementary file 2 [file Table_2.docx]

Metagenomic Analyses of Microbial and Carbohydrate-Active Enzymes in the Rumen of Holstein Cows Fed Different Forage-to-Concentrate Ratios

Lijun Wang, Guangning Zhang, Hongjian Xu, Hangshu Xin* and Yonggen Zhang*

College of Animal Science and Technology, Northeast Agricultural University, Harbin, China

^*^The first corresponding author: Yonggen Zhang and Hangshu Xin

Tel) +86-451-55190840, Fax) +86-451-55190840

E-mail: [zhangyonggen@sina.com](mailto:zhangyonggen@sina.com); [xinhangshu@163.com](mailto:xinhangshu@163.com)

**Supplement Table 2.**

**The CAZy families [glycoside hydrolases (GHs), carbohydrate esterases (CEs), pectate lyases (PLs), glycosyl Transferases (GTs), carbohydrate-binding modules (CBMs)] and other domains associated with PCWP-degrading and their abundance1 detected in four metagenomic**

| **Glycoside hydrolases (GHs)** | **Number of reads** | | | | |
| --- | --- | --- | --- | --- | --- |
|  | **LF0h** | | **LF4h** | **HF0h** | **HF4h** |
| GH0 | 94 | | 90 | 113 | 132 |
| GH1 | 24 | | 25 | 13 | 17 |
| GH10 | 205 | | 199 | 185 | 226 |
| GH103 | 9 | | 10 | 3 | 4 |
| GH105 | 392 | | 377 | 338 | 391 |
| GH106 | 154 | | 157 | 182 | 193 |
| GH108 | 10 | | 9 | 3 | 4 |
| GH109 | 38 | | 32 | 96 | 102 |
| GH11 | 2 | | 1 | 1 | 0 |
| GH110 | 0 | | 0 | 1 | 1 |
| GH113 | 1 | | 1 | 1 | 2 |
| GH115 | 332 | | 332 | 310 | 345 |
| GH116 | 2 | | 1 | 2 | 1 |
| GH117 | 4 | | 5 | 2 | 2 |
| GH120 | 2 | | 1 | 5 | 6 |
| GH123 | 5 | | 8 | 9 | 11 |
| GH124 | 1 | | 1 | 0 | 0 |
| GH125 | 54 | | 54 | 79 | 85 |
| GH127 | 168 | | 169 | 175 | 188 |
| GH128 | 2 | | 3 | 3 | 3 |
| GH13 | 795 | | 796 | 737 | 835 |
| GH130 | 145 | | 139 | 169 | 178 |
| GH16 | 140 | | 144 | 197 | 214 |
| GH18 | 72 | | 72 | 94 | 103 |
| GH19 | 7 | | 8 | 3 | 5 |
| GH2 | 1239 | | 1238 | 1272 | 1375 |
| GH20 | 126 | | 135 | 243 | 262 |
| GH23 | 227 | | 215 | 268 | 297 |
| GH24 | 57 | | 60 | 57 | 71 |
| GH25 | 335 | | 327 | 251 | 292 |
| GH26 | 166 | | 159 | 194 | 222 |
| GH27 | 74 | | 74 | 123 | 134 |
| GH28 | 564 | | 563 | 501 | 578 |
| GH29 | 211 | | 213 | 342 | 372 |
| GH3 | 1339 | | 1351 | 1669 | 1819 |
| GH30 | 103 | | 104 | 149 | 154 |
| GH31 | 515 | | 522 | 522 | 576 |
| GH32 | 256 | | 253 | 245 | 270 |
| GH33 | 86 | | 84 | 137 | 151 |
| GH35 | 129 | | 123 | 123 | 134 |
| GH36 | 171 | | 168 | 170 | 181 |
| GH37 | 1 | | 1 | 0 | 0 |
| GH38 | 2 | | 2 | 6 | 5 |
| GH39 | 6 | | 6 | 3 | 3 |
| GH4 | 1 | | 1 | 5 | 5 |
| GH42 | 13 | | 14 | 4 | 5 |
| GH43 | 1634 | | 1645 | 1703 | 1905 |
| GH44 | 1 | | 1 | 1 | 0 |
| GH45 | 1 | | 0 | 0 | 1 |
| GH48 | 0 | | 0 | 1 | 0 |
| GH5 | 638 | | 625 | 586 | 659 |
| GH50 | 0 | | 0 | 1 | 1 |
| GH51 | 475 | | 457 | 484 | 533 |
| GH53 | 262 | | 253 | 255 | 273 |
| GH54 | 9 | | 10 | 23 | 24 |
| GH55 | 1 | | 0 | 1 | 1 |
| GH57 | 160 | | 148 | 171 | 193 |
| GH59 | 0 | | 1 | 1 | 1 |
| GH63 | 7 | | 9 | 14 | 14 |
| GH65 | 3 | | 3 | 4 | 5 |
| GH66 | 16 | | 18 | 11 | 13 |
| GH67 | 194 | | 179 | 167 | 190 |
| GH73 | 73 | | 75 | 64 | 70 |
| GH76 | 13 | | 11 | 24 | 25 |
| GH77 | 98 | | 88 | 75 | 89 |
| GH78 | 243 | | 250 | 271 | 289 |
| GH8 | 118 | | 121 | 111 | 127 |
| GH84 | 6 | | 6 | 2 | 2 |
| GH88 | 35 | | 33 | 58 | 58 |
| GH89 | 65 | | 63 | 96 | 108 |
| GH9 | 154 | | 152 | 208 | 224 |
| GH91 | 1 | | 1 | 1 | 1 |
| GH92 | 257 | | 274 | 417 | 433 |
| GH93 | 6 | | 6 | 10 | 10 |
| GH94 | 147 | | 140 | 124 | 144 |
| GH95 | 341 | | 335 | 323 | 368 |
| GH97 | 605 | | 610 | 696 | 762 |
| GH99 | 1 | | 1 | 0 | 1 |
| Total | 13843 | | 13762 | 14908 | 16478 |
| **Glycosyl Transferases (GTs)** | **Number of reads** | | | | |
|  | **LF0h** | | **LF4h** | **HF0h** | **HF4h** |
| GT0 | 68 | | 67 | 90 | 112 |
| GT1 | 24 | | 20 | 4 | 8 |
| GT10 | 5 | | 5 | 9 | 9 |
| GT11 | 79 | | 78 | 64 | 79 |
| GT14 | 3 | | 3 | 16 | 18 |
| GT19 | 160 | | 161 | 195 | 206 |
| GT2 | 2775 | | 2793 | 2775 | 3089 |
| GT23 | 1 | | 2 | 1 | 1 |
| GT26 | 24 | | 22 | 34 | 38 |
| GT28 | 211 | | 213 | 218 | 236 |
| GT3 | 123 | | 123 | 118 | 132 |
| GT30 | 173 | | 176 | 153 | 179 |
| GT32 | 49 | | 55 | 82 | 84 |
| GT35 | 189 | | 193 | 196 | 208 |
| GT39 | 2 | | 0 | 0 | 0 |
| GT4 | 1147 | | 1153 | 1400 | 1558 |
| GT41 | 11 | | 11 | 0 | 0 |
| GT5 | 157 | | 162 | 155 | 176 |
| GT51 | 439 | | 430 | 433 | 487 |
| GT6 | 13 | | 12 | 10 | 11 |
| GT8 | 25 | | 24 | 23 | 26 |
| GT81 | 0 | | 0 | 1 | 1 |
| GT83 | 7 | | 7 | 15 | 15 |
| GT84 | 1 | | 1 | 1 | 1 |
| GT9 | 172 | | 175 | 161 | 176 |
| **Total** | **5858** | | **5886** | **6154** | **6850** |
|  | | | | | |
| **Carbohydrate esterases (CEs)** | | **Number of reads** | | | |
|  |  | **LF0h** | **LF4h** | **HF0h** | **HF4h** |
| CE0 | | 2 | 2 | 0 | 1 |
| CE1 | | 841 | 840 | 1148 | 1272 |
| CE10 | | 395 | 390 | 537 | 590 |
| CE11 | | 180 | 170 | 176 | 196 |
| CE12 | | 229 | 228 | 205 | 240 |
| CE13 | | 3 | 2 | 5 | 4 |
| CE14 | | 4 | 6 | 17 | 19 |
| CE15 | | 133 | 121 | 165 | 181 |
| CE2 | | 95 | 90 | 89 | 99 |
| CE3 | | 2 | 2 | 0 | 0 |
| CE4 | | 174 | 164 | 153 | 164 |
| CE6 | | 317 | 317 | 315 | 359 |
| CE7 | | 186 | 187 | 157 | 187 |
| CE8 | | 175 | 168 | 149 | 163 |
| CE9 | | 37 | 34 | 37 | 39 |
| **Total** | | **2773** | **2721** | **3153** | **3514** |
|  | |  |  |  |  |
| **Pectate**  **lyases (PLs)** | | **Number of reads** | | | |
|  |  | **LF0h** | **LF4h** | **HF0h** | **HF4h** |
| PL0 | | 7 | 9 | 16 | 15 |
| PL1 | | 392 | 373 | 332 | 363 |
| PL10 | | 107 | 105 | 105 | 120 |
| PL11 | | 214 | 198 | 210 | 231 |
| PL12 | | 0 | 1 | 2 | 2 |
| PL22 | | 2 | 2 | 2 | 2 |
| PL6 | | 1 | 1 | 0 | 0 |
| PL9 | | 29 | 29 | 33 | 32 |
| **Total** | | **752** | **718** | **700** | **765** |
|  | |  |  |  |  |
| **Carbohydrate-binding modules (CBMs)** | | **Number of reads** | | | |
|  |  | **LF0h** | **LF4h** | **HF0h** | **HF4h** |
| CBM0 | | 20 | 21 | 24 | 22 |
| CBM11 | | 1 | 1 | 1 | 1 |
| CBM12 | | 4 | 4 | 5 | 7 |
| CBM13 | | 124 | 116 | 147 | 161 |
| CBM2 | | 57 | 54 | 75 | 78 |
| CBM20 | | 211 | 208 | 191 | 213 |
| CBM22 | | 4 | 4 | 3 | 1 |
| CBM25 | | 11 | 10 | 5 | 5 |
| CBM26 | | 12 | 12 | 8 | 10 |
| CBM27 | | 2 | 2 | 0 | 1 |
| CBM3 | | 0 | 0 | 1 | 1 |
| CBM32 | | 242 | 251 | 324 | 341 |
| CBM34 | | 24 | 23 | 11 | 11 |
| CBM35 | | 58 | 54 | 67 | 76 |
| CBM37 | | 232 | 232 | 348 | 366 |
| CBM38 | | 17 | 18 | 25 | 27 |
| CBM4 | | 5 | 5 | 1 | 1 |
| CBM42 | | 6 | 9 | 19 | 20 |
| CBM48 | | 268 | 264 | 265 | 290 |
| CBM5 | | 0 | 0 | 1 | 1 |
| CBM50 | | 711 | 703 | 694 | 787 |
| CBM51 | | 16 | 19 | 43 | 42 |
| CBM57 | | 21 | 21 | 17 | 19 |
| CBM58 | | 12 | 11 | 39 | 40 |
| CBM6 | | 473 | 467 | 447 | 518 |
| CBM61 | | 17 | 16 | 7 | 10 |
| CBM62 | | 0 | 0 | 4 | 4 |
| CBM9 | | 6 | 6 | 13 | 12 |
| 总计 | | 2554 | 2531 | 2785 | 3065 |
|  | |  |  |  |  |
